# The Effect of Corticosteroids on Post-Covid-19 Smell Loss: A Meta-Analysis

**DOI:** 10.22038/IJORL.2023.72451.3456

**Published:** 2023-09

**Authors:** Mona Kabiri, Maryam Emadzadeh

**Affiliations:** 1 *Nanotechnology Research Center, Pharmaceutical Technology Institute, Mashhad University of Medical Sciences, Mashhad, Iran.*; 2 *Clinical Research Development Unit, Ghaem Hospital, Mashhad University of Medical Sciences, Mashhad, Iran*

**Keywords:** Anosmia, Hyposmia, COVID-19, Corticosteroid, Olfactory

## Abstract

**Introduction:**

The rate of olfactory loss related to COVID-19 was reported between 4-89 percent. There is no approved treatment for patients who experience anosmia after the mentioned infection. This systematic review aimed to assess the therapeutic effects of corticosteroids on anosmia in COVID-19 patients.

**Materials and Methods:**

Databases including PubMed, ISI Web of Sciences, Scopus, and Cochrane Library. Databases were searched up to September 2022 to find out randomized controlled trials that assessed the effect of corticosteroids on post-COVID anosmia/hyposmia. Only studies published in the English language were entered in this review.

**Results:**

Among the six relevant trials with a total population of 712, one study administered the combination therapy of both systemic and nasal corticosteroids, while others used intranasal corticosteroids. No significant difference was observed between the intervention (IG) and control (CG) groups in terms of duration of improvement from anosmia (mean difference:-1.799). The pooled effect of self-rating olfactory scores was assessed at 2 weeks and at the end point of the studies which revealed no significant effect in favor of the IG (pooled effect in 2 weeks: 0.739; in the endpoint: 1.32). The objective evaluation with different tools indicated that IG obtained higher scores at the endpoint of treatment. The pooled results showed that the number of patients who recovered from anosmia is higher in IG compared to CG (Odds Ratio: 1.719).

**Conclusion:**

It appears that the duration of corticosteroid therapy more than two weeks may be a considerable effect on the recovery of smell dysfunction in COVID-19 patients.

## Introduction

The COVID-19 pandemic is caused by severe acute respiratory syndrome coronavirus-2 (SARS-CoV-2) which was first found in Wuhan, China in December 2019 (1,2). 

Based on the WHO reports, the frequency of confirmed COVID-19 patients was about 603 million in the world with a mortality rate of 1.07% by September 2022 (3). Various clinical symptoms including fever, sore throat, cough, myalgia, dyspnea, and loss of smell and taste were reported in confirmed COVID-19 patients, and the severity of COVID-19 disease varied from asymptomatic to severe and critical conditions (4,5).

Although it seems that the neuroepithelium of the nasal mucosa can directly be damaged by viral infections, the olfactory loss due to COVID-19 is still in a state of ambiguity, as presented by the different range of recovery rates from 4% to 89% one month after anosmia (6-8). Previous studies revealed that COVID-19 could lead to persistent anosmia in 37-52% of patients five-week after infection (7-9). Moreover, severe long-term olfactory dysfunction was reported in 5-11.7% of COVID-19 patients, which may be more severe and prevalent relative to other viral upper airway infections (1,10,11).

Some of the comorbidities such as cognitive disorder and depression, as well as earlier mortality, may be involved in patients who suffered persistent smell loss (8,12). To date, no medical treatment was approved to recover the anosmia or hyposmia due to COVID-19 infection, and alternative therapeutic approaches are needed. Numerous studies have demonstrated the efficiency of steroid administration such as systemic or nasal corticosteroids in the treatment of anosmic or hyposmic COVID-19 patients (2,8,9,13-15).

Due to the anti-inflammatory and immunosuppressive properties of steroids, these compounds are able to block SARS-CoV-2 replication and host inflammatory responses (2,16,17). However, the immunosuppression effects of systemic corticosteroids and delayed clearance of the virus should be considered as the potential risk for secondary infections in the current pandemic (1,18-20). Some evidence revealed that systemic steroids such as dexamethasone had beneficial effects on the mortality of severe COVID-19 patients (4,21). It is necessary to mention that the advantages of systemic corticosteroid therapy may be found in the treated patients at the early stage of the COVID-19 acute phase (22). Additionally, oral corticosteroids (OCS) could improve loss of smell in some COVID-19 patients with post-viral olfactory dysfunction (PVOD) (8,23). The possible mechanism regarding the efficiency of oral steroids in mentioned patients may be related to the anti-inflammatory property in the olfactory neuroepithelium at the COVID-19 post-infectious stage (1). 

The local inflammation responses followed by the damage of the sustentacular cells may lead to anosmia development, and the permanent smell loss can be due to the progressive involvement of olfactory neurons with basal cells (24,25).

Nasal steroid therapy may be effective to combat the COVID-19 pandemic regarding the decrease of key proteins which are essential to the entrance of viral infection in the host cells, and also the down-regulation of COVID-19 replication. The mentioned process leads to decrease in inflammation reactions against viral infection (4,26,27). According to the classification of intranasal corticosteroids (INCs), the older first generation includes budesonide, flunisolide, beclomethasone, and triamcinolone, whereas the second generation of INCs such as mometasone furoate, and fluticasone (furoate or propionate) have considerably lower systemic bioavailability and adverse effects relative to other INCs, and especially OCS (28,29). 

Based on the condition of nasal mucosa secretions, the selection of INCs type is essential to change the viscosity of the nasal cavity and enhance the permeability and diffusion of steroids. Administration of ciclesonide as the hypotonic solution is recommended for dry/congested noses, while mometasone furoate and budesonide are suggested to treat patients with runny/wet noses (30). Mometasone furoate formulation has high concentrations of thixotropic agents including carboxymethylcellulose sodium and microcrystalline cellulose that enhance the viscosity of nasal mucosa and reduce moisture in the nasal cavity (28,31).

The first studies concerning the effect of systemic and intranasal corticosteroids on the recovery of olfactory dysfunction in post-COVID-19 patients have given conflicting findings. Some investigations did not recommend initial steroid therapy due to the probability of spontaneous recovery, and side effects of OCS, and INCs (1,32,33). Recent studies suggested that the corticosteroid treatment had no effect on the recovery of anosmia or hyposmia patients related to COVID-19 infection (1,4,34,35), however, some of them reported that steroids had been beneficial to recover smell loss in post-COVID-19 patients (2,9,13-15). As the mentioned controversy, the present systematic review and meta-analysis is designed to assess the efficacy of corticosteroid interventions in the treatment of post-COVID-19 olfactory loss according to published clinical trials.

## Materials and Methods

*Design*

The present meta-analysis was conducted to assess the efficacy of systemic or intranasal corticosteroid treatment on the olfactory dysfunction of patients due to COVID-19 infection. All randomized controlled trials (RCTs) regarding corticosteroid administration to recover the olfactory loss in COVID-19 patients were eligible for inclusion. This systematic review was performed in adherence to the Preferred Reporting Items for Systematic Reviews and Meta-Analyses (PRISMA).

*Search strategy*

The English-published studies were systematically searched from online databases including PubMed, ISI Web of Sciences, Scopus, and Cochrane Library up to September 2022. Searches were individually carried out by authors in various combinations using the following keywords: “Corticosteroid”, “Mometasone”, “Betamethasone”, “Budesonide”, “Ciclesonide”, “Fluticasone”, “Methylprednisolone”, “Prednisolone”, or “Dexamethasone” and “COVID-19”, “COVID”, “Coronavirus”, or SARS-Cov-2 as well as “Anosmia”, “Hyposmia”, “Microsmia”, “Olfactory” or Smell. All retrieved articles were reviewed based on the reference lists to evaluate further relevant studies. Based on the searching of registered trials, contact with the authors was accomplished to permit the use of required data in the present study.

*Study selection, data extraction, and quality assessment*

The randomized controlled trials were included in this meta-analysis and conference papers were excluded. Two authors independently checked the eligible articles through the title and abstract followed by the full text of included articles. The RCT studies were included in the meta-analysis if the effect of corticosteroids on recovery of smell loss had been evaluated in COVID-19 patients. The data extraction was conducted as followings: (a) first author’s surname, country of study, and publication year; (b) patient demographics in terms of age and gender; (c) details of trials such as the number of participants in intervention and control groups, types of corticosteroid, and duration of follow-up; and (d) outcomes of interest including assessment of objective and subjective smell loss, and frequency of recovered patients from anosmia.

Olfactory function measurements include the University of Pennsylvania smell identification test (UPSIT), the Connecticut chemosensory clinical research center (CCCRC), and the Sniffin’ Sticks test as the objective olfactory outcomes. Subjective olfactory outcomes were assessed by the Visual analog scale (VAS) and the time taken to complete recovery. The UPSIT determines the smell function of individuals based on the recognition of common odors as the score from 0 to 24. The scores categorized as anosmia (0–9), severe microsmia (10–13), mild microsmia (14–18), and the normosmia (19-24) (36). The CCCRC includes the odor identification test for common odors (10 odorants) and the butanol threshold test (9,37). Subjects recognize the odorants and fill the 20 items list including equal numbers of correct and distractor items. CCCRC test graded as anosmia (scores of 0-10), severe hyposmia (20-40), moderate hyposmia (50-60), mild hyposmia (70-80), and normal olfactory function (90-100). The visual analog scale defines olfaction by a score ranging from 0 (complete smell loss) to 10 (normal smell sensation) based on patient reports (38).

The quality assessment of the included articles was accomplished using the Jadad scale with an overall score from 0 to 5 (39). All conflicts were decided by the discussion of reviewers. A total score of 3 to 5 was considered as high-quality study.

*Statistical analysis*

The Comprehensive Meta-Analysis software (Biostat, USA) was performed, and a *P*-value < 0.05 was considered statistically significant (40). The mean difference (MD) or odds ratio (OR) with a 95% confidence interval (CI) was calculated for continuous and categorical variables, respectively. For statistical homogeneity, the mean, and standard deviation were calculated for eligible studies that reported the median with interquartile range (IQR) (41). The heterogeneity level was measured according to the inconsistency index (*I*^2^) and Cochran Q test (χ^2^). Based on the *I*^2^ index > 50% and *P*-value < 0.05, the random effects model was applied for significant heterogeneity, and the results were displayed using the forest plot. The source of heterogeneity was assessed by sensitivity and sub-group analysis. The Egger’s linear regression test was also used to report the publication bias.

## Results

*Summary of searches*

**Fig 1:**
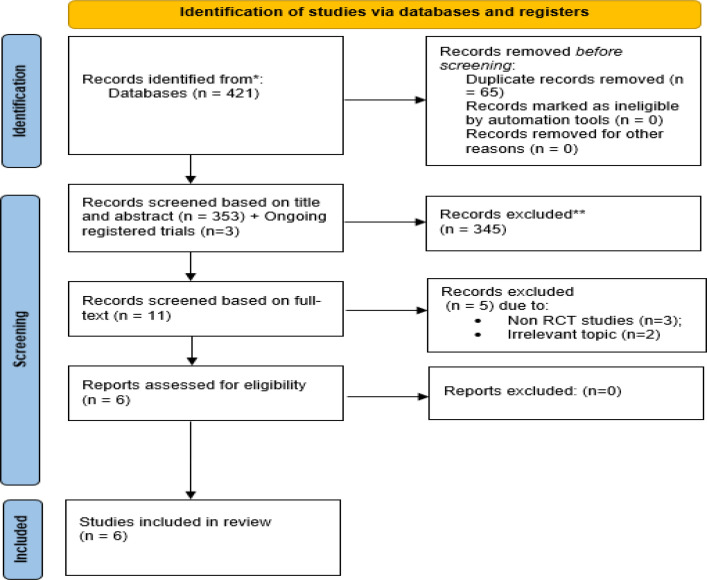
The PRISMA flow diagram

After searching the mentioned databases, 421 studies were found in the first step. The number of mentioned studies was reduced to 356 by excluding duplicate articles. The title and abstract of these articles were reviewed by the authors to rule out the irrelevant ones. Three of them were ongoing registered trials without published articles (42-44). Finally, eleven articles were assessed by full-text, and by removing five of them (1, 3-5, 13), six relevant randomized controlled trials were evaluated in the present systematic review (2,9,14,15,34, 35) (Figure 1).

*Risk of bias*

The quality of each study was assessed using the Jadad score. Most of the included studies counted as high quality and only one study received one score which was categorized as low quality (15). In this study, there was no description regarding the exact randomization, nor drop-out. The details of the quality of the articles are shown in Table 1.

**Table 1:** Quality assessment of the included studies

**ID**	Was the study described as randomized?*	Was the study described as a double blind?*	Was there a description of withdrawal and dropouts?*	The randomization scheme described and appropriate*	The method of double blinding described and appropriate*	The randomization scheme described and inappropriate**	The method of double blinding described and inappropriate**	**Total score**
Kasiri H, 2021	1	1	1	1	0	0	0	4
Abdelalim A.A, 2021	1	0	1	1	0	0	0	3
Hosseinpour M, 2022	1	1	1	1	0	0	0	4
Rashid RA, 2021	1	1	1	0	1	0	0	4
Vaira LA, 2020	1	0	1	1	1	0	0	4
Yildiz E, 2021	1	0	0	0	0	0	0	1

* Yes: +1; No: 0, ** Yes: -1; No: 0

*Characteristics of included studies*

Table 2 demonstrates the data of each study and their results in detail. From six eligible studies, two were based in Iran (2,14), one in Turkey (15), one in Iraq (35), one in Egypt (34), and one in Italy (9). All studies (the total population = 712 (were conducted on adults at least 18 years old. Male to female ratio was less than one in four of included studies (2,9,34,35). 

The population of the Hosseinpour and Vaira studies had chronic microsmia/anosmia (more than 30 days) (2,9), while in other studies the patients suffered from acute odor disorders.

 All of the studies administered the nasal spray of corticosteroids, whereas one study administered a combination steroid therapy including systemic prednisolone and nasal irrigation with betamethasone (9). The type of intranasal spray was mometasone furoate in three studies (2,14, 34), betamethasone in two studies (9,35), and triamcinolone in one study (15).

*Outcomes*

**Fig 2:**
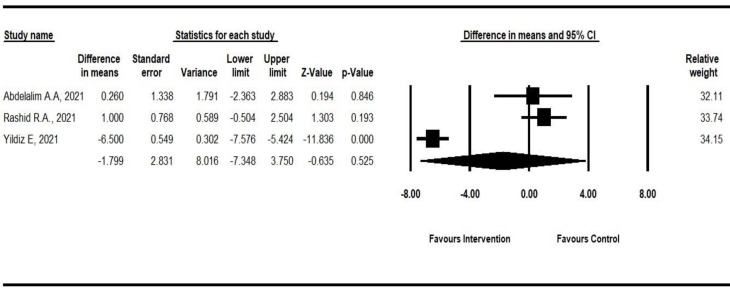
Forest plot of the duration of improvement from anosmia in intervention and control groups

Outcomes are assessed in three main categories of *subjective outcomes* (duration of improvement from anosmia and a self-rating olfactory score), *objective outcome*s, and the *frequency of recovered patients from anosmia after an intervention*. As shown in figure 2, three studies mentioned the *olfactory dysfunction duration* in anosmic or hyposmic patients (15, 34, 35). Only Yildiz et al. indicated that the duration of improvement from anosmia is significantly lower in the intervention group (mean difference: -6.500; 95%CI: -7.576 to -5.424; p-value < 0.001). According to the pooled result, no significant difference was observed between the intervention and control groups (mean difference: -1.799; 95%CI: -7.348 to 3.75; p-value: 0.525; *I*^2^:97.22).

**Fig 3:**
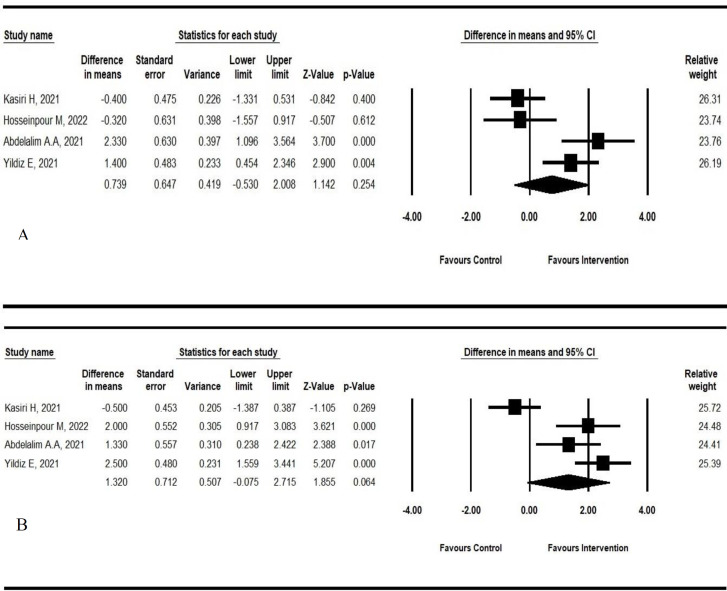
Forest plot of the Self-rating olfactory scores in intervention and control groups: a) at 2 weeks; b) at the endpoint of the study

*Self-rating olfactory scores* were assessed in four studies (2,14,15,34) at different time points. We pooled the results of this score at 2 weeks and the endpoints of the studies (figure 3a, b). 

The results revealed that the intervention at 2 weeks was significantly effective in two studies (15,34), whereas at the endpoint the meaningful consequence of corticosteroids was seen in three studies (2,15,34) (pooled effect in 2 weeks: 0.739; 95%CI: -0.53 to 0.008; p-value: 0.254; *I*^2^:82.11; pooled effect in the endpoint: 1.32; 95%CI: -0.075 to 2.715; p-value: 0.064; *I*^2^:87.28). Smell test changes as an objective evaluation conducted in three studies with different methods (2,9,14) Kasiri et al. and Hosseinpoor et al. applied IRAN-SIT (as the modified standardized version of UPSIT based on Iranian cultural features) for the evaluation of anosmia, however, Vaira used the CCCRC test in this regard. In all three mentioned studies, the intervention group (IG) obtained higher scores at the endpoint of treatment compared to the control groups (CG). There was no significant between-group difference based on IRAN-SIT in the two mentioned studies (mean difference: 1.922; 95%CI: 1.319 to 5.163; p-value: 0.245). This difference was not statistically significant in Kasiri et al. study at 4 weeks (mean change in IG: 8.1±5.1; in CG: 7.9±5; p-value: 0.91) and Hosseinpoor et al. at 2 weeks (mean change in IG: 3.31±2.53; in CG: 2.88±2.81; p-value: 0.05) of intervention.

These changes were reported as 10.8±4.22 and 6.57±3.62 at 4 weeks in intervention and control groups, respectively in the study conducted by Hosseinpoor (p-value<0.001). Vaira reported the median (IQR) of CCCRC at baseline, 20 days, and 40 days of the study. At baseline, the median (IQR) of CCCRC was higher in the control group (p-value: 0.586), while in both two points of follow-up, the intervention group scored significantly higher compared to the control (p-value: 0.011 at 20 days, and p-value: 0.024 for 40 days) (Table 2).

**Table 2:** Characteristics of the included studies

**Author/year**	**Country**	**Population**	**Intervention (type, dose, sample size, duration)**	**Control (type, sample size)**	**Duration of follow-up**	**Outcome**	**Results**
Kasiri H, 2021	Iran	Non-hospitalized adult patients with COVID-19 (≥18 years) who had severe microsmia or anosmia within two weeks; Mean age (SD) in IG:35.4±2; CG:33.2±8.5); M/F ratio: 1.02	Mometasone furoate 0.05% nasal spray at a dose (100 μg) twice daily in each nostril for 4 weeks along with olfactory training; (n=40)	Two puffs of topical saline spray in each nostril twice daily together with olfactory training for 4 weeks; (n=40)	4 weeks	* Frequency of patients who return to normosmia $ The improvement of the olfactory score based on the "Visual analog scale (VAS) score (0-10) # Iran Smell Identification Test (Iran-SIT) score (0-24) at the end of the study	* 48.7% in IG; 21.1% in CG $ Mean change of VAS at 1^st^ week (IG:2.1±1.9; CG:2.4±1.7) 2^nd^ week (IG: 4±2.4; CG:4.4±1.7) 3^rd^ week (IG:4.8±2.2; CG:5.3±1.6) 4^th^ week (IG:5.2±2.3; CG:5.7±1.6) # Mean (SD) of Iran-SIT changes in IG:8.1±5.1; CG: 7.9±5
Abdelalim A.A, 2021	Egypt	Non-hospitalized adults (>18 years) who recently recovered from COVID-19 and sudden recent anosmia/hyposmia with or without loss of taste; Median (IQR) age in IG: 28 (20.5-38); in CG: 30 (22.5-39); M/F ratio: 0.85	Mometasone furoate nasal spray in the dose of 2 puffs (100 μg) once daily in each nostril for 3 weeks with olfactory training; (n=54)	Olfactory training; (n=54)	3 weeks	* Frequency of fully recovered patients § The duration of anosmia/hyposmia till full recovery (days) $ The improvement of the olfactory score based on the "Visual analog scale (VAS) score	* 62% in IG; 52% in CG § IG: 26.41±7.99 vs CG: 26.15±5.07 $ Median (IQR) at baseline (IG:2(0.5-5);CG: 2(0.5-5)) 1^st^ week (IG:5(2-5); CG:2(1-5)) 2^nd^ week (IG:7(5-10);CG:5(2-8)) 3^rd^ week (IG:10(9-10); CG:10(5-10))
Hosseinpoor M, 2022	Iran	Non-hospitalized adult (>18 years) COVID-19 patients who had persistent anosmia or severe microsmia between 30-90 days; Mean age (SD) in IG: 32.23±10.02; CG: 34.93±12.39; M/F ratio: 0.55	One puff of 0.05% W/V mometasone furoate intranasal spray on each side twice per day for 4 weeks; (n=40)	One puff of 0.65% W/V sodium chloride nasal spray on each side twice daily for 4 weeks; (n=40)	4 weeks	* Frequency of patients who return to normosmia $ The improvement of the olfactory score based on the "Visual analog scale (VAS) score # Iran Smell Identification Test (Iran-SIT) score (0-24) at the endpoint	* 54.3% in IG; 42.8% in CG $ Mean change of VAS at 2^nd^ week (IG: 1.73±1.55; CG:2.05±2.5) 4^th^ week (IG:4.66±2.36; CG:2.66±2.26) # Mean (SD) of Iran-SIT changes at 2 weeks (IG: 3.31±2.53; CG:2.88±2.81); at 4 weeks (IG:10.08±4.22; CG: 6.57±3.62)
Rashid RA, 2021	Iraq	Outpatient mild to moderate COVID-19 with the recent development of anosmia (≥ 18 years); Median (IQR) age in IG: 29 (23-37); in CG: 29 (23-35); M/F ratio: 0.40	intranasal betamethasone sodium phosphate drops (0.1 mg/ mL) 3 drops for each nasal cavity 3-times daily until recovery for a maximum of one month; (n=138)	placebo drops (0.9% NaCl solution) 3-times daily until recovery for a maximum of one month; (n=138)	One month	* Frequency of patients who recovered from anosmia within the follow-up period § Time taken for anosmia to resolve (days)	* 82% in IG; 84% in CG § Median (IQR) of ODD (IG: 7(5-14); CG: 7(4-12))
Vaira LA, 2020	Italy	Adults (≥ 18 years) with mild to moderate COVID-19-related anosmia or severe hyposmia (CCCRC test score ≤ 40) for more than 30 days; Mean age (SD) in IG: 42.5±9; in CG: 41.5±9.1); M/F ratio: 0.64	systemic prednisone (starting with 1 mg/ kg/day and tapering the dose for 15 days) & Nasal irrigation with betamethasone, ambroxol, a mucolytic, and rinazine, a decongestant, for 15 days; (n=9)	Untreated (Receive no drug); (n=9)	40 days	* Frequency of patients recovered from anosmia # CCCRC test	* 55% in IG; 0% in CG # Median (IQR) of CCCRC test changes at 20^th^ day (IG:40(45); CG: 10(15)); at 40^th^ day (IG: 60(40); CG: 30(25))
Yildiz E, 2021	Turkey	Admitted patients with confirmed COVID-19 and acute odor loss; Mean age (SD) in IG: 37.2±8.4; in Group 1: 38.5±10.5; in Group 2: 39.2±11.3; M/F ratio: 1.27	saline irrigation (hypertonic solution/10 cc per nose, twice a day/1 month) and nasal steroid spray (2*2 puffs/each nose/Triamcinolone Acetonide 0.055%); (n=50)	Group 1: Not given any extra treatment (untreated); (n=50)	One month	§ Olfactory Dysfunction Duration (ODD) on the 1^st^, 15^th,^ and 30^th^ days (face-to-face interviews on the first day, and a telephone survey on the other days) $ Self-Rating Olfactory Score (SROS)	§ Mean±SD of ODD: IG:5.6±3.2 vs. CG:12.1±2.2 $ Mean (SD) of SROS at baseline: IG:2.7±3.3; Group1:3.1±2.5; Group 2: 2.8±2.4; 15^th^ day: IG:6.1±2.5; Group1:4.1±2.6; Group2:4.8±3.1; 30^th^ day: IG 8.5±3.2; Group1:5.2±2.3; Group2:6.1±2.2
				Group 2: saline irrigation (hypertonic solution/10 cc to each nose, twice a day/1 month) for treatment; (n=50)			

CCCRC: Connecticut chemosensory clinical research center; IG: Intervention Group; CG: Control Group. The endpoint VAS score was reported at three weeks in the Abdelalim study and four weeks for others. The frequency of recovered patients from anosmia was based on an objective or subjective test performed in each RCT.

**Fig 4:**
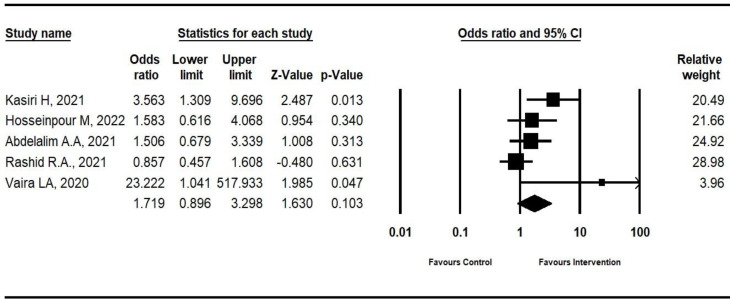
Forest plot of the frequency of recovered patients from anosmia

The number of patients who completely recovered from anosmia was reported in five studies (2,9,14,34,35). The results favor intervention in every single study, except the one conducted by Rashid et al. The pooled results indicated that the corticosteroid may have effective results in increasing the number of improved patients from anosmia (Odds Ratio: 1.719; 95%CI: 0.896 to 3.298; p-value: 0.103; *I*^2^:54.99), although one of them demonstrated significant result (14). Figure 4 displays the results of each study as well. 

*Heterogeneity analysis*

To assess the sources of heterogeneity, subgroup, and sensitivity analysis were conducted. 

According to the sources of heterogeneity, the sub-group analysis was applied based on chronic (2,9) or acute anosmia (14,15,34,35); as well as topical (2, 14,15,34,35), or combination therapy with systemic corticosteroids (9); and utilizing administration in the absence (2,9,15,35) or presence (14,34) of olfactory training. Detailed results are presented in Table 3. Self-rating olfactory scores were significantly increased in the intervention group compared to the control group in those who received only corticosteroids as an intervention (without olfactory training) (2,15) after 4 weeks.

**Table 3:** Subgroup analysis of the interest outcomes in included studies

**Variables**	**Chronicity**		**Type of corticosteroids**		**Corticosteroid therapy**	
	**Chronic**	**Acute**	**Topical**	**Topical+Systemic**	**With olfactory training**	**Without olfactory training**
Frequency of recovered patients^*^	3.96 (0.32, 48.2); p:0.27	1.53 (0.70, 3.34); p:0.27	1.51 (0.85, 2.67); p:0.15	23.22 (1.04, 517.93); p:0.04^**^	2.19 (0.94, 5.05); p:0.06	1.48 (0.53, 4.13); p:0.44
VAS 2 weeks	-0.32 (-1.55, 0.91); p:0.61^**^	1.4 (-0.48, 2.62); p:0.17	-		0.93 (-1.74, 3.6); p:0.49	0.58 (-1.09, 2.27); p:0.49
VAS 4 weeks	2 (0.91, 3.08); p<0.001^**^	1.1 (-0.71, 2.92); p:0.23	-		0.38 (-1.4, 2.17); p:0.67	2.28 (1.57, 2.99); p<0.001
Duration of improvement	-		-		0.26 (-2.36, 2.88); p:0.84^**^	-2.76 (-0.11,4.58); p:0.46

*This data was reported as Odds ratio (95%CI), while in other variables the data were reported as difference in means (95%CI). ** These results were reported only in one single study.

As shown in figure 4, the study conducted by Vaira et al. (9) had the lowest relative weight. However, the sensitivity analysis indicated that by removing this study, the final pooled OR did not change much (Odds Ratio: 1.511; 95% CI: 0.852 to 2.679; p-value: 0.158) (data not shown). Also, Egger’s linear regression test was used to assess the publication bias which showed no evidence of publication bias (p=0.06) for the outcome (the number of completely recovered patients) which included the five RCTs.

## Discussion

In the current systematic review and meta-analysis, the findings of all controlled trials administered corticosteroids on patients with COVID-19-related anosmia were pooled. No significant difference was found concerning the effect of corticosteroid therapy versus control on the decrease of anosmia duration. There were no significant effects in favor of intervention in terms of the self-rating olfactory score (SROS) at 2 weeks or 4 weeks of follow-up. Although most of the included studies illustrated that the number of patients who completely recovered from anosmia was higher in the intervention group relative to the control, the meta-analysis did not reveal any specific effect in favor of corticosteroids.

Among the six included studies, Yildiz et al. indicated that the time taken for anosmia to resolve in the intervention group is approximately one week less than the two control groups (15). Noted that the placebo-controlled group (group 2) was selected for pooling the quantitative outcomes such as VAS and duration of recovery in this study. The type of nasal steroid in this study was triamcinolone, however, in two other studies, mometasone furoate and betamethasone were administered and their results were in favor of the control (34, 35). A prospective interventional study conducted by Singh et al. reported that administration of both fluticasone nasal spray and triamcinolone paste could improve odor loss and taste within a week, in Indian patients who suffered from both anosmia and dysgeusia, (13). Using the mentioned combination therapy did not determine whether the fluticasone spray itself was effective or the combined treatment. Also, as noted by Verma, the lack of a standardized objective testing tool is one limitation of the mentioned research (45). In another study, Clemency et al. reported that the median duration of all COVID-19-related symptoms to recovery was 19 days in both ciclesonide and placebo groups (4). 

Five studies reported the frequency of completely recovered patients from anosmia (2, 9,14,34,35) in which the odds ratio of corticosteroid in four of them favors corticosteroid (2,9,14,34). Vaira with only nine participants in the intervention and nine in the placebo arm, reported the highest odds ratio, while the confidence interval was the widest (9). Keep in mind that this study was the only one that used both systemic and intranasal corticosteroids and it was also one of the two studies which investigated the effect of corticosteroids on chronic anosmia. 

Another significant result belonged to the study by Kasiri et al., which reported about 50% of participants who received mometasone furoate with olfactory training (OT), completely experienced normosmia after the intervention period versus 21% of the placebo group recovered. (14). In contrast, Clemency et al. presented that the frequency of recovered patients was 70% and 63% in the ciclesonide and placebo groups recovered, respectively (4). Moreover, Singh reported that the response to fluticasone spray treatment is different in terms of the kind of smell such as 83% response rate to mint smell and 93% to musky smell on the fifth day after intervention (13). According to the data collected from 3,191 asymptomatic-to-mild COVID-19 patients in Korea, acute anosmia or ageusia was reported in approximately 15% of patients and was predominant in females and young adults (46). Lee et al. reported that most of them recovered within three weeks and the median time to recover from anosmia was about a week (46). Additionally, Kim D.H. et al. reported no statistically significant difference in olfactory recovery between intervention and control groups. This study only reviewed the efficacy of topical corticosteroids in acute olfactory loss (37). In the present review, half of the studies which reported olfactory scores based on VAS showed a significant improvement in this score after two weeks (15,34). Over time, one more study indicated an improvement in this score (2), whereas Kasiri et al. did not report any significant change after one month. All of these studies were performed on those with acute odor loss.

The methods used to assess olfactory dysfunction were different according to the included studies. Although some studies reported subjective evaluation of olfactory scores based on the visual analog scale (14,38), others used smell pens (Sniffin’ Sticks tests) (1, 47,48) or smell bottles (CCCRC) (9,37). The scores of objective assessment in the current review were higher in all intervention groups relative to the control group, while this difference was only significant in more than 20 days of follow-up. Hosseinpoor study at 4 weeks and Vaira et al. at 20 and 40 days of follow-up found this difference in favor of the intervention (2,9).

Although Kasiri et al. suggested that the administration of mometasone furoate therapy with olfactory training could enhance the rate of recovery relative to olfactory training, the mean change of VAS and UPSIT did not differ between the two groups. Additionally, the mentioned study did not report the UPSIT score at two weeks of follow-up.

In our review, two studies used olfactory training in combination with corticosteroids as an intervention (14,34). However, these studies compared the effect of this combination with olfactory training as the control group, and the effectiveness of OT alone is not clearly determined. Using Olfactory training as the treatment of odor loss was mentioned in some studies with different consequences. Miwa et al. (2019) reviewed literature published between 1990 and 2014 to find out an evidence-based recommendation for the approach to olfactory dysfunction (22). They concluded that although no single drug is beneficial for post-viral olfactory dysfunction, olfactory training could be effective in improving olfactory function. Moreover, the systematic review by Hura et al. recommended olfactory training for post-viral olfactory dysfunction (12). 

This review also noted that the administration of corticosteroids in PVOD could be attended after precise consideration of steroid risks (12). Le Bon et al. reported the results of the administration of oral corticosteroids to patients with persistent dysosmia. Their results demonstrated that the changes in threshold discrimination identification (TDI) score were higher in those who received both oral corticosteroid and OT compared to OT alone group (8). The results of the current review may be influenced by the severity of the previous COVID-19 infection. Although most of the included studies investigated adults (> 18 years) who had a history of mild to moderate COVID-19, the severity of the previous COVID-19 disease was not mentioned in the study conducted by Kasiri et al. (14). Moreover, Abdelalim noted that some of the included patients might have a history of hospitalization according to COVID-19 infection (34). It should be altered that the frequency of female patients in all included studies was slightly more than males except in the study performed by Yildiz et al., and Kasiri et al. (14,15).

## Conclusion

In the present systematic review and meta-analysis, the efficacy of corticosteroid treatments was evaluated in the anosmic or hyposmic patients related to COVID-19 infection. Among six included studies, five RCTs compared nasal corticosteroid therapy with control, whereas one study assessed both systemic and intranasal treatment as the intervention group. Additionally, two studies evaluated olfactory loss in chronic anosmia or microsmia patients, and other trials were performed in acute patients. Our results revealed no significant differences in terms of objective and olfactory tests as well as the frequency of recovered patients from anosmia between intervention and control groups. 

Although the number of relevant studies was small, three trials illustrated the significant effects of corticosteroids after two weeks of intervention based on the self-rating olfactory scores. It seems that the duration of treatment more than two weeks may be a significant effect on the recovery of olfactory dysfunction due to COVID-19.
